# Diagnostic value of quantitative SPECT/CT integrated parameters for differentiating spinal tuberculosis from spinal metastases

**DOI:** 10.3389/fonc.2026.1890572

**Published:** 2026-07-30

**Authors:** Jiaxing Wang, Zhiwei Ma, Xingyu Duan, Yingqin Jia, Yuxin Gao, Qian Zhao, Jiong Wang, Ningkui Niu

**Affiliations:** 1 Department of Orthopedics, General Hospital of Ningxia Medical University, Yinchuan, China; 2 The First Clinical Medical College of Ningxia Medical University, Yinchuan, China; 3 Department of Orthopedics, The Third People’s Hospital of Yinchuan City, Yinchuan, China; 4 Department of Nuclear Medicine, General Hospital of Ningxia Medical University, Yinchuan, China; 5 Key Laboratory of Clinical Pathogenic Microorganisms, General Hospital of Ningxia Medical University, Yinchuan, China; 6 Research Center for Prevention and Control of Bone and Joint Tuberculosis, General Hospital of Ningxia Medical University, Yinchuan, China

**Keywords:** CT attenuation, quantitative SPECT/CT, spinal metastases, spinal tuberculosis, standardized uptake value

## Abstract

**Objectives:**

To evaluate the diagnostic value of quantitative ^99m^Tc-MDP SPECT/CT metabolic, CT attenuation, and adjacent vertebra integrated parameters for differentiating spinal tuberculosis (STB) from spinal metastases (SM).

**Materials and methods:**

This retrospective study included 212 patients who underwent quantitative ^99m^Tc-MDP SPECT/CT, including 94 with STB and 118 with SM. Volumes of interest (VOIs) were delineated on fused SPECT/CT images, from which standardized uptake value (SUV), total bone uptake (TBU), metabolic bone volume (MBV), and CT Hounsfield unit (HU) parameters were obtained. One target metastatic vertebra was analyzed in each SM patient, whereas paired adjacent involved vertebrae were used to construct integrated parameters in STB patients. Diagnostic performance was assessed using ROC analysis, multivariable logistic regression, DeLong’s test, and decision curve analysis.

**Results:**

SUVmax, SUVmean, metabolic bone volume, tracer bone uptake, CT HUmax, and CT HUmean were significantly higher in SM target vertebrae than in STB involved vertebrae, all *p* < 0.001. Among single integrated parameters, the lesion burden index based on SUVmax (LBI_SUVmax_) showed the best performance, with an AUC of 0.854, sensitivity of 95.7%, and specificity of 73.7%. The AUCs of the metabolic, CT attenuation, and combined models were 0.938, 0.810, and 0.943, respectively. DeLong’s test showed that the combined model outperformed the CT attenuation model, LBI_SUVmax_, but not the metabolic model, *p* = 0.422.

**Conclusion:**

Quantitative ^99m^Tc-MDP SPECT/CT provides objective information for noninvasive differentiation of STB and SM, and metabolic integrated parameters may offer useful diagnostic value.

## 1 Introduction

Spinal tuberculosis (STB) and spinal metastases (SM) are two clinically important spinal disorders with distinct etiologies, treatment strategies, and prognostic implications. STB is an infectious granulomatous disease caused by *Mycobacterium tuberculosis* and commonly involves contiguous vertebral bodies, the intervertebral disc, and adjacent paravertebral soft tissues (1). In contrast, SM usually results from hematogenous dissemination of malignant tumors and may present as isolated or multifocal vertebral lesions, often with relative preservation of the intervertebral disc and without abscess formation (2). Despite these different pathological bases, STB and SM may show overlapping clinical and imaging manifestations, including axial pain, neurological deficits, vertebral destruction, and paravertebral soft-tissue changes, making preoperative differentiation challenging (3). Conventional imaging modalities have important limitations in this setting. Magnetic resonance imaging (MRI) is the main imaging tool for evaluating spinal lesions because it can depict bone marrow involvement, disc space abnormalities, epidural extension, and paravertebral soft-tissue changes (4). However, its specificity remains limited when infection-related inflammatory changes and tumor infiltration show overlapping signal characteristics, particularly when typical features of STB are absent (5). Computed tomography (CT) provides detailed information on cortical destruction, sclerosis, sequestra, and local bone architecture, but it has limited ability to evaluate early marrow-based lesions and cannot directly reflect functional bone metabolism (6). Conventional three-phase technetium-99m methylene diphosphonate (^99m^Tc-MDP) bone scintigraphy is sensitive for detecting increased osteoblastic activity, but its specificity is limited because degenerative changes, post-traumatic remodeling, and other benign skeletal conditions may also show increased tracer uptake (7, 8).

Quantitative single-photon emission computed tomography/computed tomography (SPECT/CT) with ^99m^Tc-MDP enables the integrated assessment of functional bone metabolic activity and CT-based anatomical information, providing a non-invasive approach for quantitative evaluation of spinal lesions (9). Previous studies have shown that SPECT/CT can improve lesion localization and diagnostic assessment in infectious spinal disorders, thereby providing useful information for clinical evaluation and treatment planning (10, 11). In spinal metastases, quantitative parameters such as the maximum standardized uptake value (SUVmax) and mean standardized uptake value (SUVmean) can reflect lesion-related bone metabolic activity and may facilitate the identification of suspected osseous metastatic lesions (12). In addition, radiomics studies based on SPECT/CT images have also demonstrated the potential value in differentiating vertebral metastases from benign vertebral lesions (13).

However, important gaps remain in the current literature. Quantitative SPECT/CT studies of spinal and skeletal lesions have mainly focused on differentiating benign from malignant bone lesions or on detecting and characterizing metastatic bone disease (14). Currently, studies using quantitative SPECT/CT parameters to differentiate STB from SM remain limited (15). In particular, few studies have incorporated into quantitative parameter construction the characteristic pattern of STB, which often spreads contiguously through the intervertebral disc and along the subligamentous region beneath the anterior longitudinal ligament, typically involving two adjacent vertebral bodies. Therefore, the main objective of this study is to evaluate the quantitative ^99m^Tc-MDP SPECT/CT parameters, including metabolic parameters and CT attenuation parameters, in terms of their diagnostic value for differentiating spinal tuberculosis from spinal metastases. Additionally, it aims to further explore the potential value of the integrated parameters and diagnostic models constructed based on the adjacent affected vertebrae of spinal tuberculosis.

## 2 Materials and methods

### 2.1 Study population

This retrospective study was approved by the Institutional Review Board of the General Hospital of Ningxia Medical University and was conducted in accordance with the Declaration of Helsinki. The requirement for written informed consent was waived because anonymized routinely acquired clinical and imaging data were used. No animal experiments were performed. Consecutive patients who underwent quantitative ^99m^Tc-MDP SPECT/CT at the General Hospital of Ningxia Medical University between September 2024 and March 2026 were retrospectively reviewed. Eligible patients were identified through the electronic medical record system and institutional imaging database. A total of 212 patients met the inclusion criteria, including 94 patients with STB and 118 patients with SM. For quantitative analysis, patients were enrolled at the patient level. In the SM group, one selected target metastatic vertebra was analyzed for each patient. The target vertebra was selected according to the biopsy site when available, corresponding clinical symptoms, the level of neural compression, and concordant MRI, CT, and SPECT/CT findings, rather than according to SUV or CT HU values. In the STB group, two adjacent involved vertebrae were used to construct an integrated analysis unit, according to the characteristic pattern of contiguous vertebral involvement in STB.

### 2.2 Inclusion and exclusion criteria

The inclusion criteria were as follows: (1) patients with STB confirmed by microbiological, molecular, or histopathological evidence, including positive acid-fast bacilli smear or culture, molecular testing such as Xpert MTB/RIF, targeted next-generation sequencing positive for the Mycobacterium tuberculosis complex, or histopathological examination of biopsy specimens; (2) patients with SM who had a known primary malignancy confirmed by histopathology and spinal metastatic lesions confirmed by conventional imaging and SPECT/CT; (3) completion of diagnostic-quality 99mTc-MDP SPECT/CT suitable for quantitative analysis; (4) availability of complete clinical, laboratory, and conventional imaging data.

Patients were excluded if they met any of the following criteria: (1) spinal infection other than STB, including pyogenic spondylodiscitis, fungal spondylitis, brucellar spondylitis, or polymicrobial infection; (2) primary spinal tumors or hematologic malignancies involving the spine; (3) local treatment before SPECT/CT, including radiotherapy, surgery, vertebroplasty, or other interventions that could substantially affect local bone metabolism or structural morphology; (4) incomplete clinical data, incomplete conventional imaging data, or SPECT/CT images unsuitable for quantitative analysis.

### 2.3 Image acquisition and analysis

All included patients underwent ^99m^Tc-MDP SPECT/CT according to routine clinical protocols. Imaging was performed using a Symbia Intevo Bold SPECT/CT system (Siemens Healthineers). ^99m^Tc-MDP was prepared using ^99m^Tc-pertechnetate eluted from a ^^99Mo/^99m^Tc generator (Atom High Tech Co., Ltd.) and an MDP kit (Jiangsu Institute of Nuclear Medicine). After intravenous injection of 20 to 25 mCi ^99m^Tc-MDP, patients were instructed to drink water and void before imaging. Image acquisition was performed approximately 3 h after tracer injection. No iodinated or gadolinium based contrast agent was administered during SPECT/CT. Patients were placed in the supine position. Whole body bone scintigraphy was first performed, followed by regional SPECT/CT acquisition centered on the site of abnormal tracer uptake.

The SPECT acquisition parameters were as follows. The photopeak was set at 140 keV with a 20% energy window, a 256 × 256 matrix, 6° per frame, 20 s per frame, and a total of 30 frames. CT was performed using a 16 slice spiral CT scanner with a tube voltage of 130 kV, tube current of 160 mA, and a 512 × 512 matrix. To ensure quantitative accuracy, patient height, body weight, radioactivity in the syringe before and after injection, corresponding measurement times, injection time, and scan time were recorded. SUVs were normalized to body weight. The net injected activity was calculated as the radioactivity in the syringe before injection minus the residual radioactivity after injection, with physical decay correction applied according to the corresponding measurement times, injection time, and scan time. SUV was calculated as follows:


$$SUV=\frac{Activity\;concentration\;within\;the\;VOI\;(kBq/mL)}{Injected\;activity\;(kBq)/Body\;weight\;(kg)}$$

**Figure 1:**
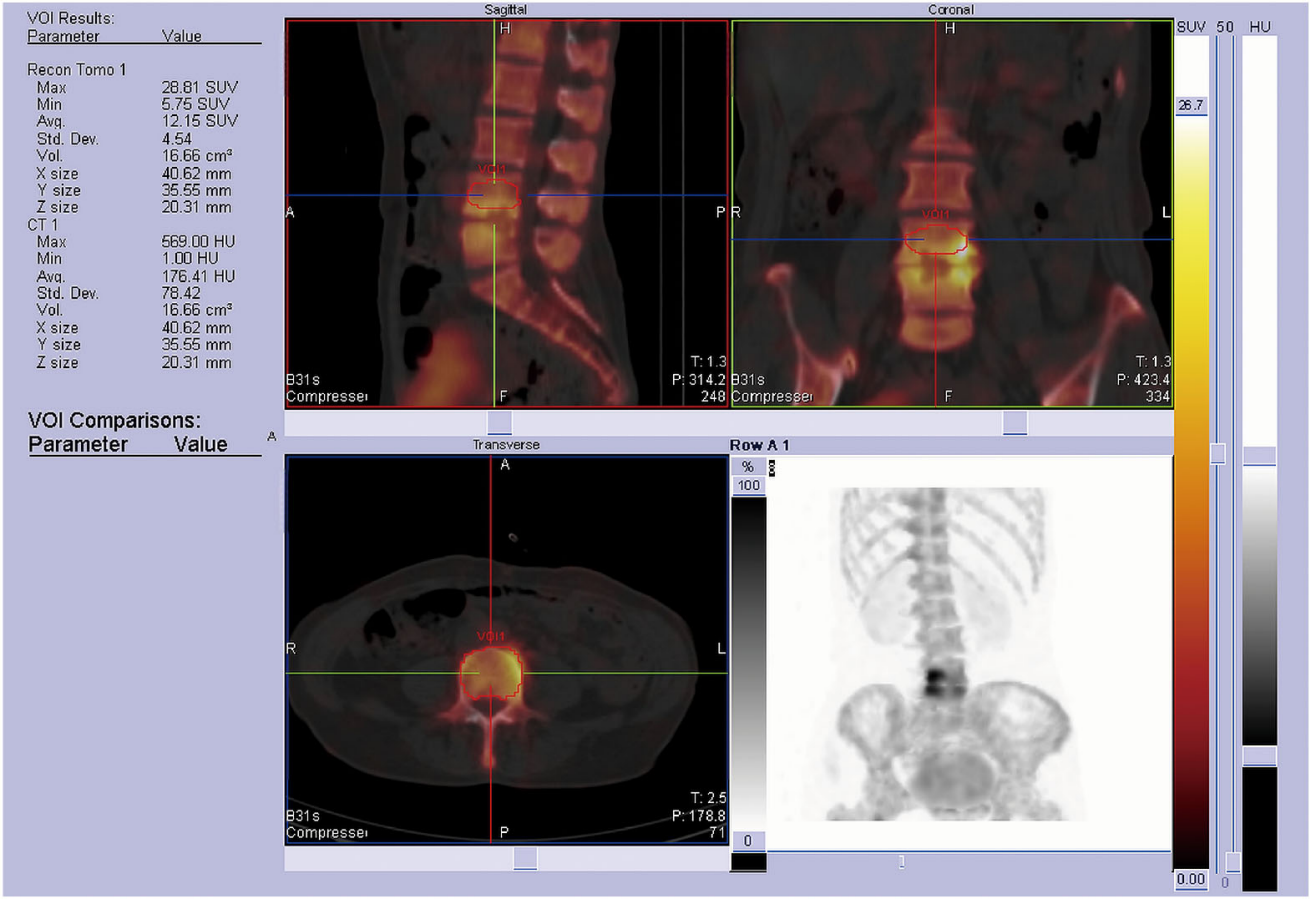
Quantitative single-photon emission computed tomography/computed tomography (SPECT/CT) examination obtained in a patient with spinal tuberculosis involving the L4 and L5 vertebra. Fused SPECT/CT images in the sagittal, coronal, and axial planes, together with the corresponding planar bone scintigraphy image, demonstrate increased radiotracer uptake in the involved L4 and L5 vertebral lesion.

**Figure 2:**
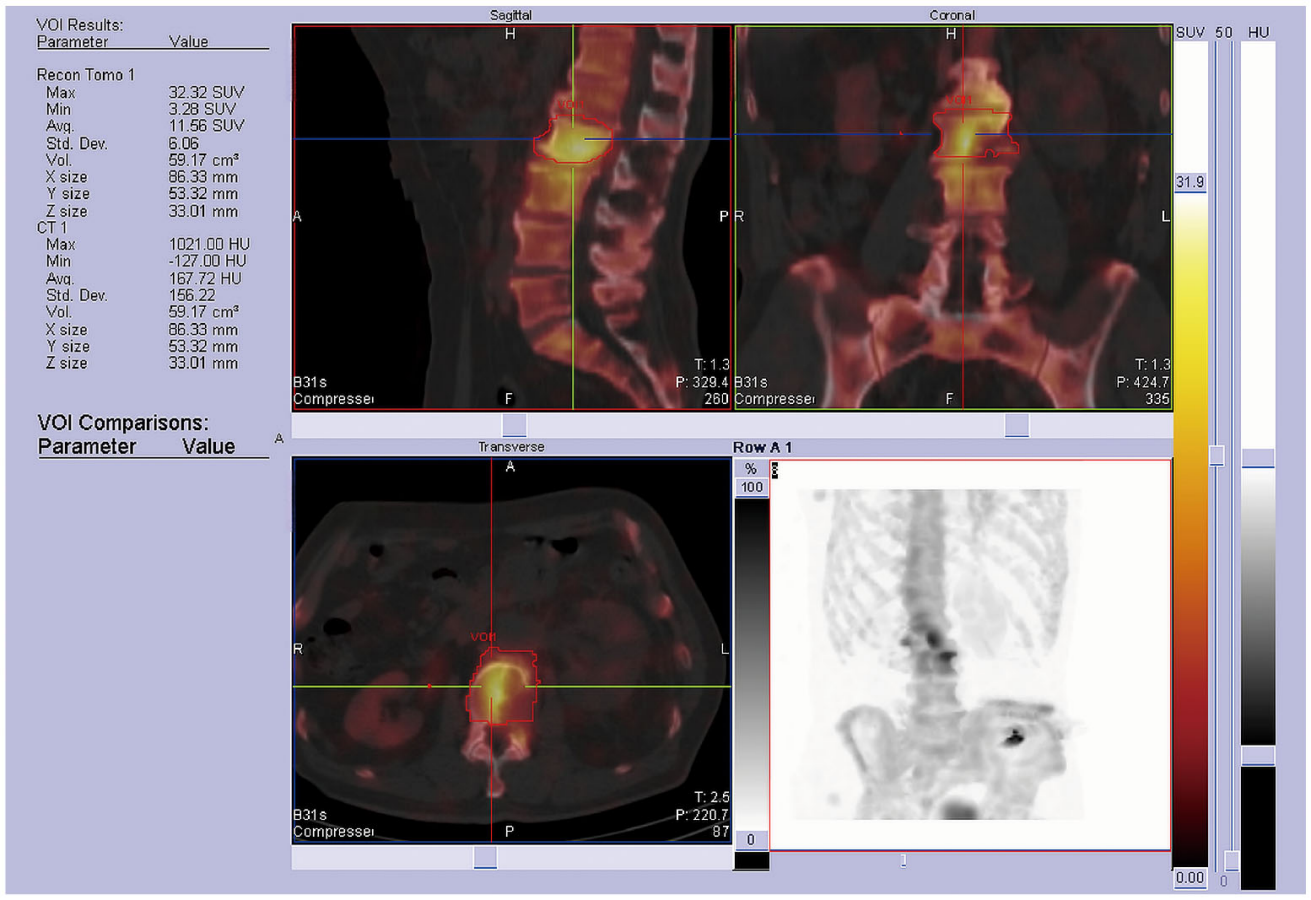
Quantitative single-photon emission computed tomography/computed tomography (SPECT/CT) examination obtained in a patient with spinal metastases involving the L2 vertebra. Fused SPECT/CT images in the sagittal, coronal, and axial planes, together with the corresponding planar bone scintigraphy image, demonstrate increased radiotracer uptake in the involved L2 vertebral lesion.

Image post processing was performed on a Syngo workstation using Esoft software (Siemens Healthineers). SPECT images were reconstructed using the ordered subset expectation maximization algorithm with 2 iterations and 10 subsets, together with CT based attenuation correction, scatter correction, and resolution recovery. Abnormal tracer-avid vertebral lesions were identified on fused SPECT/CT images. After the target vertebrae or adjacent involved vertebrae had been determined, volumes of interest (VOIs) were delineated on fused SPECT/CT images. VOIs were generated using semi-automatic segmentation followed by manual correction. The VOIs mainly covered the abnormal tracer-avid and corresponding osseous involved area within the selected vertebra, rather than the entire vertebral body. Adjacent normal vertebrae, intervertebral discs, paravertebral soft tissues, and clearly non-lesional structures were excluded. Metabolic parameters and CT attenuation parameters were extracted from the same co-registered VOIs. See **Figures 1** and **2**.

For the SM group, one target metastatic vertebra was selected for each patient to maintain consistency of the analysis unit. When pathological sampling of a spinal lesion was available, the vertebra corresponding to the sampling site was selected. Otherwise, the target vertebra was selected based on clinical symptoms, the level of neural compression, and concordant MRI, CT, and SPECT/CT findings. For the STB group, considering the typical involvement of adjacent vertebrae in spinal tuberculosis, quantitative parameters from paired adjacent involved vertebrae were integrated according to predefined rules to generate derived imaging parameters for subsequent statistical analysis and model construction. All SPECT/CT examinations used for quantitative analysis were performed before spinal biopsy or surgical sampling, and target-vertebra selection was completed before quantitative parameter measurement rather than according to SUV or CT HU values. All images were jointly reviewed by two experienced nuclear medicine physicians, and final results were recorded after consensus was reached.

### 2.4 Quantitative parameter collection and construction of integrated parameters

Patient demographic and examination-related data were recorded, including sex, age, height, body weight, body mass index (BMI), and the time interval between radiotracer injection and xSPECT acquisition. Quantitative SPECT/CT parameters were obtained from the volumes of interest, including metabolic parameters and CT attenuation parameters. The metabolic parameters included the maximum standardized uptake value (SUVmax), minimum standardized uptake value (SUVmin), mean standardized uptake value (SUVmean), total bone uptake (TBU), and metabolic bone volume (MBV). The CT attenuation parameters included the maximum, minimum, and mean computed tomography Hounsfield unit values (CT HUmax, CT HUmin, and CT HUmean, respectively).

Considering the typical contiguous involvement of adjacent vertebrae in spinal tuberculosis, and the single or multiple vertebral involvement pattern of spinal metastases, different quantitative analysis strategies were used for the two groups. In the SM group, the original quantitative parameters from one selected target metastatic vertebra in each patient were used for analysis. In the STB group, quantitative parameters from two adjacent involved vertebrae in each patient were integrated according to predefined rules. The integrated metabolic parameters constructed for the STB group included lesion burden index based on SUVmax (LBI_SUVmax_), integrated SUVmin (SUVmin_int_), lesion burden index based on SUVmean (LBI_SUVmean_), and arithmetic mean of TBU (TBU_AM_). The integrated CT attenuation parameters included the lesion burden index based on CT Humax (LBI_CT Humax_), integrated CT HUmin (CT HUmin_int_), and lesion burden index based on CT HUmean (LBI_CT HUmean_). Specifically, LBI_SUVmax_, LBI_SUVmean_, LBI_CTHUmax_​ and LBI_CTHUmean_ were calculated using metabolic bone volume-weighted integration to reflect the relative contribution of each involved vertebra to the overall lesion volume. SUVmin_int_ and CT HUmin_int_ were defined as the lower values between the two adjacent involved vertebrae, aiming to capture the low-uptake or low-attenuation component within the contiguous lesion. TBU_AM_ represented the arithmetic mean of total bone uptake across the two adjacent involved vertebrae. For each STB patient, the two selected adjacent involved vertebrae were ordered anatomically, with the cranial vertebra defined as vertebra 1 and the caudal vertebra as vertebra 2. Variables with the subscript “1” and “2” therefore represent measurements from the cranial and caudal involved vertebrae, respectively. The formulas for these parameters are provided below.


$$LBI_{suvmax}=\frac{SUVmax_{1}\times MBV_{1}+SUVmax_{2}\times MBV_{2}}{MBV_{1}+MBV_{2}}$$

$$SUVmin_{int}=\min(SUVmin_{1},SUVmin_{2})$$

$$LBI_{suvmean}=\frac{SUVmean_{1}\times MBV_{1}+SUVmean_{2}\times MBV_{2}}{MBV_{1}+MBV_{2}}$$

$$TBU=SUV_{mean}\times MBV$$

$$TBU_{AM}=\frac{TBU_{1}+TBU_{2}}{2}$$

$$LBI_{CTHUmax}=\frac{CTHUmax_{1}\times MBV_{1}+CTHUmax_{2}\times MBV_{2}}{MBV_{1}+MBV_{2}}$$

$$CT\;HUmin_{int}=\min(CT\;HUmin_{1},CT\;HUmin_{2})$$

$$LBI_{CTHUmean}=\frac{CTHUmean_{1}\times MBV_{1}+CTHUmean_{2}\times MBV_{2}}{MBV_{1}+MBV_{2}}$$

### 2.5 Statistical analysis

Statistical analyses were performed using Python software, version 3.12.7, and GraphPad Prism, version 10.6. Continuous variables were expressed as the mean ± standard deviation for normally distributed data or as the median with interquartile range [median (IQR); 25th–75th percentiles] for non-normally distributed data. Between-group comparisons were performed using the independent-samples *t* test or the Mann–Whitney *U* test, as appropriate. Categorical variables were expressed as counts and percentages and compared using the χ² test or Fisher’s exact test.

The main diagnostic performance analysis was based on the STB adjacent-vertebra integrated analysis unit and the SM target-vertebra analysis unit. Multivariable logistic regression was used to construct the metabolic parameter model, CT attenuation parameter model, and combined model. Variables included in the multivariable logistic regression models were selected according to predefined imaging-parameter categories and model parsimony, rather than solely on the basis of univariable analysis. The metabolic parameter model included the integrated metabolic parameters, and the CT attenuation parameter model included the integrated CT attenuation parameters. The combined model included metabolic and CT attenuation parameters that remained independently associated with the diagnostic outcome in the corresponding category-specific models. Multicollinearity among variables in the multivariable logistic regression models was assessed using variance inflation factors (VIFs). Receiver operating characteristic (ROC) curves were used to evaluate the diagnostic performance of individual quantitative parameters and multivariable models. The area under the curve (AUC), optimal cutoff value, sensitivity, specificity, positive predictive value (PPV), negative predictive value (NPV), and Youden index were calculated. DeLong’s test was used for pairwise comparison of AUCs between correlated ROC curves. With STB defined as the positive class, decision curve analysis was performed using model-predicted probabilities to evaluate the net benefit of each model across threshold probabilities from 0.01 to 0.80. A two-sided *p* < 0.05 was considered statistically significant.

## 3 Results

### 3.1 Baseline characteristics and quantitative analysis units

A total of 212 patients with confirmed spinal lesions were included in this study, including 94 patients in the STB group and 118 patients in the SM group. No significant differences were observed between the two groups in age, sex, body weight, height, body mass index, or the time interval between tracer injection and xSPECT acquisition, all *p* > 0.05, as shown in **Table 1**. In the SM group, lung cancer was the most common primary malignancy, followed by prostate cancer and breast cancer. Other primary tumor types are listed in **Supplementary Table 1**.

**Table 1:** Baseline characteristics and quantitative analysis design of the STB and SM groups.

Variable	STB group (n = 94)	SM group (n = 118)	*P* value
Baseline clinical characteristics			
Age, years	57.91 ± 10.50	60.00 ± 8.97	0.128
Sex, male/female	45/49	52/66	0.677
Weight, kg	55.50(48.75,61.00)	52.60(48.10,59.78)	0.129
Height, m	1.67 ± 0.08	1.65 ± 0.06	0.059
BMI, kg/m2	19.96 ± 3.124	19.73 ± 3.034	0.644
Injection-to-xSPECT interval, min	207.10 ± 26.40	210.30 ± 27.20	0.392
Lesion characteristics and quantitative analysis design			
Patients included, n	94	118	
Vertebrae included in original-parameter comparison, n	188 involved vertebrae	118 selected target vertebrae	
Primary analysis unit	Adjacent involved vertebral pair	Selected target metastatic vertebra	
No. of primary analysis units	94	118	
Single-vertebra involvement in the SM group, n (%)		35(29.7)	
Multiple-vertebra involvement in the SM group, n (%)		83(70.3)	

Primary analysis units were adjacent involved vertebral pairs in the STB group and selected target metastatic vertebrae in the SM group.

**Figure 3:**
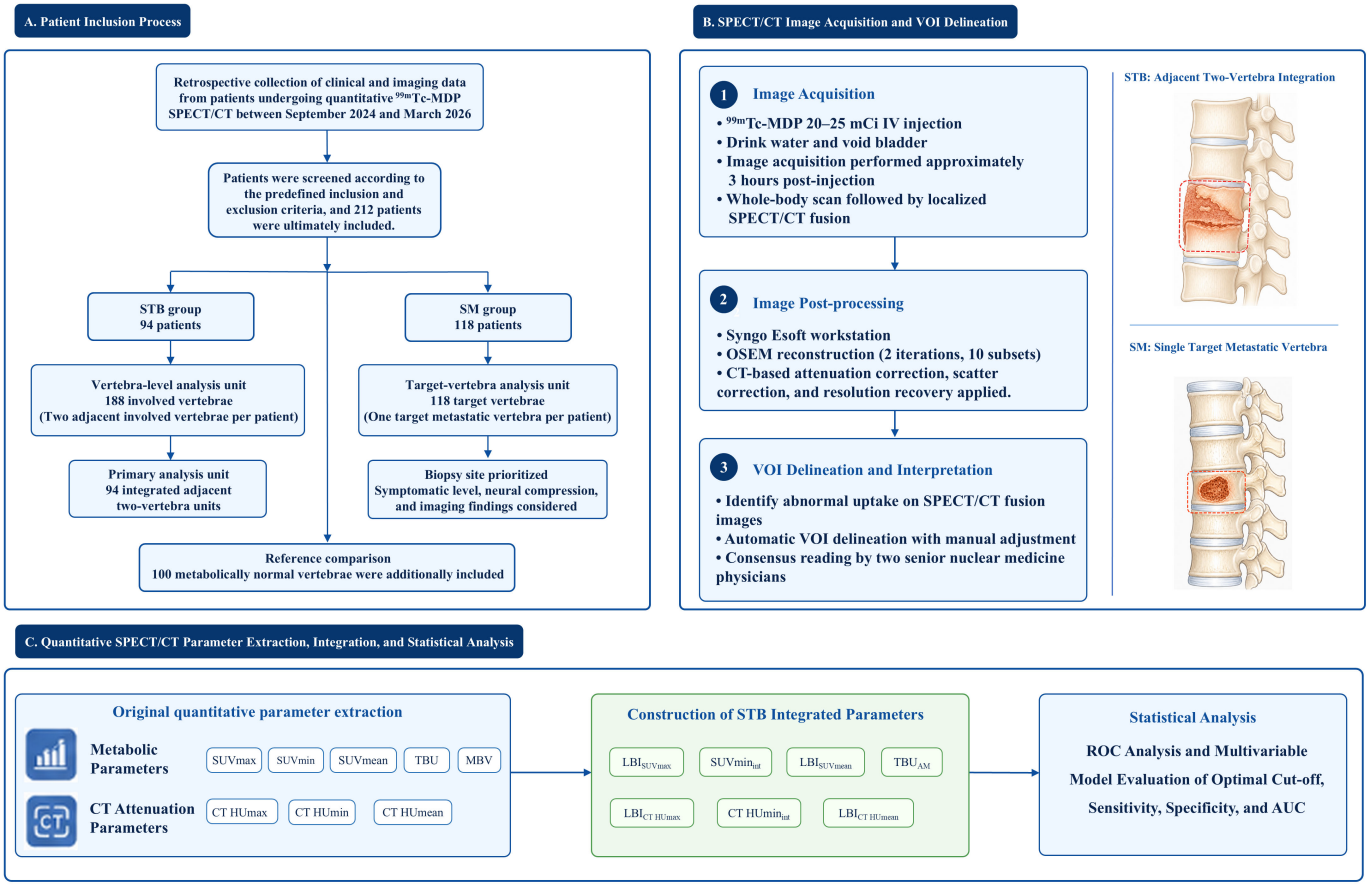
Workflow for patient inclusion and quantitative SPECT/CT analysis of vertebral lesions. **(A)** Patient inclusion and construction of the STB, SM, and reference normal groups. **(B)** SPECT/CT image acquisition, image reconstruction, volume-of-interest delineation, and the quantitative analysis units used for STB and SM. **(C)** Extraction of original quantitative SPECT/CT parameters, construction of STB integrated parameters, and statistical analysis.

For the analysis units, 188 adjacent involved vertebrae from the STB group were included for vertebra-level comparison of original parameters and were further integrated into 94 adjacent-vertebra analysis units. In the SM group, 118 target metastatic vertebrae were included according to the principle of selecting one target vertebra per patient for original parameter comparison and subsequent main analyses. Among patients with SM, 35 patients had single-vertebra involvement, accounting for 29.7%, and 83 had multiple-vertebra involvement, accounting for 70.3%. In addition, 100 metabolically normal vertebrae were included as reference normal vertebrae for comparison of original quantitative parameters. Baseline characteristics and analysis unit composition are shown in **Table 1**, and the patient inclusion and quantitative analysis workflow is shown in **Figure 3**.

### 3.2 Comparison of original vertebra-level quantitative SPECT/CT parameters

Original vertebra-level quantitative SPECT/CT parameters were compared among STB-involved vertebrae, SM target metastatic vertebrae, and reference normal vertebrae, as shown in **Table 2**. Among the three groups, significant overall differences were observed in SUVmax, SUVmean, MBV, TBU, CT HUmax, CT HUmin, and CT HUmean, all *p* < 0.001, whereas SUVmin showed no significant difference. Among these parameters, SUVmax, SUVmean, MBV, and TBU were higher in SM target metastatic vertebrae than in STB-involved vertebrae and reference normal vertebrae.

**Table 2:** Comparison of original vertebra-level quantitative SPECT/CT parameters among STB-involved vertebrae, selected SM target vertebrae, and reference normal vertebrae.

Variable	STB-involved vertebrae (n = 188)	Selected SM target vertebrae (n = 118)	Reference normal vertebrae (n = 100)	Over all *P* value	STB vs. SM
Metabolic parameters					
SUVmax	17.33 (15.20,19.15)	25.55 (20.55,30.85)	7.13 (6.65,8.12)	0.001	0.001
SUVmin	3.77 (3.16,4.36)	3.79 (3.04,4.61)	3.75 (3.52,4.14)	0.719	0.759
SUVmean	9.16 (7.75,10.61)	12.74 (9.44,16.16)	5.60 (4.91,6.53)	0.001	0.001
MBV	25.68 (18.86,32.37)	29.17 (24.25,35.05)	21.07 (16.32,27.06)	0.001	0.001
TBU	232.9 (159.9,316.7)	359.3 (251.6,489.0)	126.9 (81.2,176.3)	0.001	0.001
CT attenuation parameters					
CT HU max	809.5 (734.3,928.5)	932.0 (824.5,1065.0)	883.0 (838.5,945.0)	0.001	0.001
CT HU min	-25.0 (-35.0, -15.0)	-24.0 (-29.0, -17.0)	-49.00 (-59.0, -43.2)	0.001	0.669
CT HU mean	259.0 (210.3,301.8)	330.0 (276.0,366.0)	187.5 (168.0,216.0)	0.001	0.001

STB-involved vertebrae refer to the 188 affected vertebrae recorded in the STB group, whereas selected SM target vertebrae refer to one target metastatic vertebra selected from each patient in the SM group.

**Figure 4:**
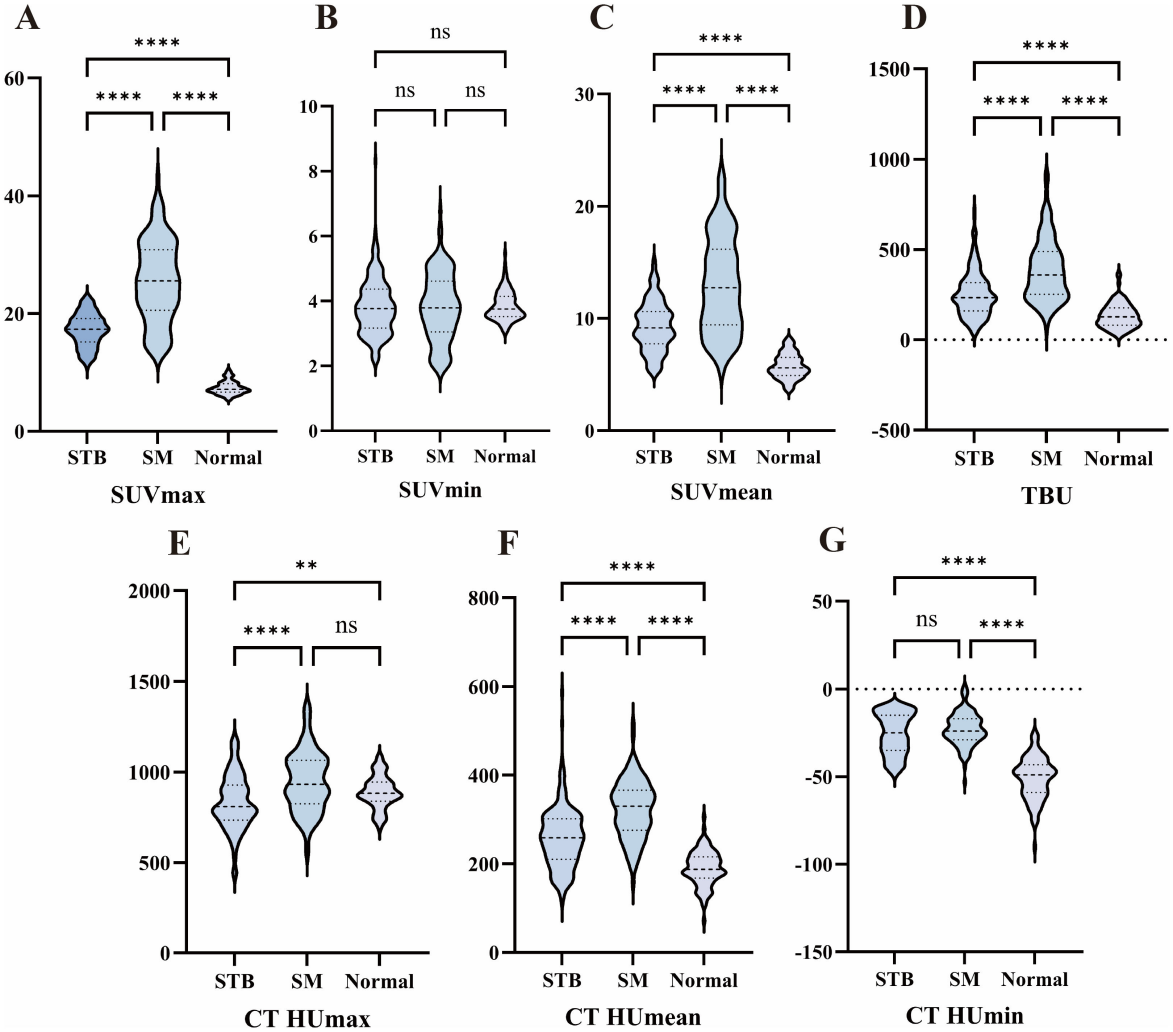
Quantitative SPECT/CT parameter distributions among STB-involved vertebrae, selected SM target vertebrae, and reference normal vertebrae: **(A)** SUVmax; **(B)** SUVmin; **(C)** SUVmean; **(D)** TBU; **(E)** CT HUmax; **(F)** CT HUmean; and **(G)** CT HUmin. **p < 0.01; ****p < 0.001; ns, not significant.

In the pairwise comparison between STB-involved vertebrae and selected SM target metastatic vertebrae, data are presented as the median (IQR; 25th–75th percentiles). The SM group showed higher SUVmax [25.55 (20.55, 30.85) vs. 17.33 (15.20, 19.15)], SUVmean [12.74 (9.44, 16.16) vs. 9.16 (7.75, 10.61)], MBV [29.17 (24.25, 35.05) vs. 25.68 (18.86, 32.37)], and TBU [359.3 (251.6, 489.0) vs. 232.9 (159.9, 316.7)], all p < 0.001. In contrast, SUVmin showed no significant difference between the two groups, *p* = 0.759. For CT attenuation parameters, selected SM target metastatic vertebrae also showed higher CT HUmax [932.0 (824.5, 1065.0) vs. 809.5 (734.3, 928.5)] and CT HUmean [330.0 (276.0, 366.0) vs. 259.0 (210.3, 301.8)], both p < 0.001, whereas CT HUmin showed no significant difference between the two groups, *p* = 0.669, as shown in **Figure 4**.

### 3.3 Comparison of STB integrated parameters with corresponding parameters of SM target vertebrae

Quantitative SPECT/CT parameters were further compared between the STB adjacent-vertebra integrated analysis units and the SM target vertebrae, as shown in **Table 3**, data are presented as the median (IQR; 25th–75th percentiles). For metabolic parameters, LBI_SUVmax_, SUVmin_int_, LBI_SUVmean_, and TBU_AM_ in the STB group were all significantly lower than the corresponding SUVmax, SUVmin, SUVmean, and TBU values in the SM group, all *p* < 0.001. The most pronounced difference was observed for LBI_SUVmax_, with a median value of 17.50 (15.48, 19.30) in the STB integrated analysis units compared with 25.55 (20.55, 30.85) for SUVmax in the SM target vertebrae. Among CT attenuation parameters, LBI_CTHUmean_ showed a representative difference between the two groups, with lower values in the STB integrated analysis units than in the SM target vertebrae [258.5 (220.5, 293.3) vs. 330.0 (276.0, 366.0), p = 0.001]. Other integrated metabolic and CT attenuation parameters showed similar trends and are summarized in **Table 3**.

**Table 3:** Comparison of STB integrated quantitative SPECT/CT parameters with corresponding original parameters in SM target vertebrae.

Category	STB integrated parameter	STB integrated analysis units (n = 94)	SM target parameter	SM target vertebrae (n = 118)	*P* value
Integrated metabolic parameters	LBI_SUVmax_	17.50 (15.48,19.30)	SUVmax	25.55 (20.55,30.85)	0.001
	SUVmin_int_	3.25 (2.92,3.92)	SUVmin	3.79 (3.04,4.61)	0.001
	LBI_SUVmean_	9.30 (8.10,10.39)	SUVmean	12.74 (9.44,16.16)	0.001
	TBU_AM_	229.9 (178.9,332.7)	TBU	359.3 (251.6,489.0)	0.001
Integrated CT attenuation parameters	LBI_CT HUmax_	797.0 (742.5,916.3)	CT HUmax	932.0 (824.5,1065.0)	0.001
	CT HUmin_int_	-30.0 (-40.00,-20.00)	CT HUmin	-24.00 (-29.0,-17.0)	0.001
	LBI_CT HUmean_	258.5 (220.5,293.3)	CT HUmean	330.0 (276.0,366.0)	0.001

Integrated parameters were calculated only for the STB group using paired adjacent involved vertebrae, whereas SM values represent original parameters measured in selected target metastatic vertebrae.

### 3.4 Diagnostic performance of individual quantitative parameters

The ROC analysis results for the original parameters of the cranial and caudal involved vertebrae in the STB group and the corresponding parameters of the SM target vertebrae are shown in **Supplementary Table 3**. Among metabolic parameters, SUVmax showed relatively high diagnostic performance, with AUCs of 0.8419 for the cranial vertebra and 0.8679 for the caudal vertebra. The AUCs of SUVmean were 0.7490 and 0.7687, and those of TBU were 0.6624 and 0.8173, respectively. SUVmin showed relatively low diagnostic performance, with AUCs of 0.5324 and 0.5533. Among CT attenuation parameters, CT HUmean showed relatively high diagnostic performance, with AUCs of 0.7522 and 0.7796 for the cranial and caudal vertebrae, respectively.

The ROC analysis results for the STB adjacent-vertebra integrated parameters and the corresponding parameters of the SM target vertebrae are shown in **Table 4**. Among integrated metabolic parameters, LBI_SUVmax_ had the highest AUC, at 0.8542, with a sensitivity of 95.74%, specificity of 73.73%, and Youden index of 0.695. The AUCs of LBI_SUVmean_, TBUAM, and SUVmin_int_ were 0.7590, 0.7468, and 0.6327, respectively. Among integrated CT attenuation parameters, LBI_CT HUmean_ had the highest AUC, at 0.7694, whereas the AUCs of LBI_CT HUmax_ and CT HUmin_int_ were 0.7091 and 0.6839, respectively.

**Table 4:** Diagnostic performance of integrated STB parameters in differentiating STB from SM target vertebrae.

Variable	AUC (95% CI)	Cutoff	Sensitivity (%)	Specificity (%)	PPV (%)	NPV (%)	Youden index
STB Integrated metabolic parameters							
LBI_SUVmax_	0.8542 (0.8011–0.9074)	21.27	95.74	73.73	74.38	95.6	0.695
SUVmin_int_	0.6327 (0.5582–0.7072)	4.44	91.53	35.11	63.91	76.74	0.266
LBI_SUVmean_	0.7590 (0.6946–0.8234)	12.52	96.81	51.69	61.49	95.31	0.485
TBU_AM_	0.7468 (0.6824–0.8113)	413.0	95.74	40.68	56.25	92.31	0.364
STB Integrated CT attenuation parameters							
LBI_CT HUmax_	0.7091 (0.6400–0.7781)	884.0	71.28	64.41	61.47	73.79	0.357
CT HUmin_int_	0.6839 (0.6097–0.7581)	-34.0	43.62	88.98	75.93	66.46	0.326
LBI_CT HUmean_	0.7694 (0.7058–0.8329)	294.0	76.60	66.95	64.86	78.22	0.435

STB integrated parameters were derived from paired adjacent involved vertebrae and compared with corresponding original parameters of selected SM target vertebrae.

### 3.5 Comparison of diagnostic performance among multivariable models

The metabolic parameter model achieved an AUC of 0.9381, with 95% confidence intervals (CIs) of 0.9000–0.9762. The sensitivity, specificity, PPV, NPV, and Youden index were 82.98%, 90.68%, 87.64%, 86.99%, and 0.737, respectively. In this model, LBI_SUVmax_ and SUVmin_int_ showed independent associations, with odds ratios (ORs) of 0.4075 and 44.52, respectively. Although SUVmin_int_ showed limited diagnostic performance in single-parameter ROC analysis, with an AUC of 0.6327, it remained independently associated with the diagnostic outcome in the metabolic model. The CT attenuation model had an AUC of 0.8095, with a 95% CI of 0.7500–0.8690. Its sensitivity, specificity, PPV, NPV, and Youden index were 65.96%, 79.66%, 72.09%, 74.60%, and 0.456, respectively. LBI_CT HUmax_, CT HUmin_int_, and LBI_CT HUmean_ also showed independent associations in the CT attenuation model.

The combined model included LBI_SUVmax_, SUVmin_int_, LBI_CT HUmax_, CT HUmin_int_, and LBI_CT HUmean_. Among the three multivariable models, the combined model achieved the highest AUC and Youden index, with an AUC of 0.9427, a 95% CI of 0.9051–0.9803, and a Youden index of 0.830. The sensitivity, specificity, PPV, and NPV were 87.23%, 95.76%, 94.25%, and 90.40%, respectively. In the combined model, LBI_SUVmax_ and SUVmin_int_ remained independently associated with the diagnostic outcome, whereas the 95% CIs of the ORs for LBI_CT HUmax_, CT HUmin_int_, and LBI_CT HUmean_ crossed 1. The diagnostic performance of the multivariable models is summarized in **Table 5**, and the regression coefficients, ORs, and VIFs are provided in **Supplementary Table 2**.

**Table 5:** Diagnostic performance of multivariable models in differentiating STB from SM target vertebrae.

Model	AUC (95% CI)	Sensitivity (%)	Specificity (%)	PPV (%)	NPV (%)	Youden index
Metabolic model	0.9381 (0.9000–0.9762)	82.98	90.68	87.64	86.99	0.737
CT attenuation model	0.8095 (0.7500–0.8690)	65.96	79.66	72.09	74.60	0.456
Combined model	0.9427 (0.9051–0.9803)	87.23	95.76	94.25	90.40	0.830

The metabolic model included LBI_SUVmax_, SUVmin_int_, LBI_SUVmean_, and TBU_AM_. The CT attenuation model included LBI_CT HUmax_, CT HUmin_int_, and LBI_CT HUmean_. The combined model included LBI_SUVmax_, SUVmin_int_, LBI_CT HUmax_, CT HUmin_int_, and LBI_CT HUmean_. Classification metrics were calculated using a probability threshold of 0.50, with STB considered the positive class.

DeLong’s test was used to further compare the AUCs of the ROC curves, as shown in **Supplementary Table 4**. The AUC of the combined model was significantly higher than those of the CT attenuation model, LBI_SUVmax_, and LBI_CT HUmean_. The metabolic model also showed a significantly higher AUC than the CT attenuation model. However, no significant difference was observed between the combined model and the metabolic model. Decision curve analysis showed that the combined model and the metabolic model provided greater net benefit than the CT attenuation model across the prespecified threshold probability range, with relatively similar net benefit curves between the two models.

## 4 Discussion

STB and SM are clinically important spinal disorders with overlapping clinical symptoms and conventional imaging findings, but they require markedly different treatment strategies (16). Accurate preoperative differentiation is therefore essential for guiding anti-tuberculosis therapy, oncological management, surgical planning, and biopsy decisions. Although image-guided biopsy remains an important method for establishing the pathological nature of spinal lesions, non-diagnostic or false-negative results may occur because of sampling location, sample volume, lesion heterogeneity, or prior treatment (17, 18). Therefore, objective and non-invasive imaging biomarkers may provide useful adjunctive information for differentiating STB from SM.

Quantitative ^99m^Tc-MDP SPECT/CT enables simultaneous assessment of functional bone metabolic activity and CT-based structural attenuation (19). The uptake of ^99m^Tc-MDP and other phosphonate-based bone tracers is mainly related to local bone turnover, osteoblastic activity, and blood perfusion (20, 21). Increased bone remodeling, vascular dilation, or elevated regional blood flow within a lesion may lead to increased ^99m^Tc-MDP accumulation, thereby providing more objective quantitative information than visual assessment alone (22). Yoo et al. reported that quantitative SPECT/CT-derived SUV parameters could be used to assess bone metastatic burden and assist in the identification of bone metastases in prostate cancer (23). Recent studies have also explored the use of quantitative bone SPECT/CT in infectious bone diseases (24). However, in spinal infection, especially STB, existing research still mainly focuses on MRI and CT morphological features, and studies on standardized quantitative SPECT/CT parameter systems remain limited (25).

In this study, selected SM target vertebrae generally showed higher metabolic parameters and CT attenuation values than STB-involved vertebrae. This difference may be related to the distinct pathological mechanisms of the two diseases. In SM, tumor-cell infiltration and tumor-mediated cytokine activity can induce bone destruction, reactive bone remodeling, osteoblastic activity, and increased regional blood perfusion, thereby increasing 99mTc-MDP uptake. Depending on the primary tumor type, metastatic lesions may also show osteoblastic, osteolytic, or mixed structural changes, which can influence CT attenuation parameters (26). It also suggests that morphological assessment alone may be insufficient to accurately distinguish these two types of lesions, whereas quantitative metabolic parameters can provide additional information for differential diagnosis (27).

Previous quantitative SPECT/CT studies have mostly used single lesions or selected target vertebrae as the analysis unit, focusing mainly on tracer uptake levels or differences between benign and malignant bone lesions (28). Few studies have incorporated disease specific lesion distribution patterns into parameter construction. STB often involves adjacent vertebrae and may show contiguous spread along the intervertebral disc, subligamentous region, or adjacent structures (29). SM is commonly related to hematogenous dissemination of malignant tumors to the spine and may present as isolated or multiple vertebral metastases (30). Given these different involvement patterns, comparison at the single vertebra level may not fully reflect the contiguous involvement pattern of STB. Therefore, this study integrated metabolic activity and CT attenuation information from two adjacent involved vertebrae in STB to construct integrated parameters based on adjacent vertebrae, aiming to reflect the overall lesion burden of continuously involved vertebrae and to make the quantitative analysis more consistent with the disease specific imaging characteristics of STB and SM.

Further comparison between the integrated parameters and the original parameters of the cranial and caudal involved vertebrae showed that adjacent vertebra integration in STB did not simply repeat single vertebra information. SUVmin_int_ and CT HUmin_int_ achieved AUCs of 0.6327 and 0.6839, respectively, which were higher than the AUC ranges of their corresponding original parameters, with SUVmin ranging from 0.5324 to 0.5533 and CT HUmin ranging from 0.5224 to 0.5632, as shown in **Table 4** and **Supplementary Table 3**. This improvement may be related to the minimum value integration strategy. By selecting the lower value from two adjacent involved vertebrae, these parameters emphasize the low uptake and low attenuation components within continuously involved STB lesions. Because spinal tuberculosis is often accompanied by caseous necrosis, bone destruction, and inflammatory changes, metabolic activity and bone density may be unevenly distributed within the lesion. Low value parameters may therefore better reflect infectious destruction and enhance the quantitative difference between STB and SM target vertebrae (31). For the other integrated SPECT/CT parameters, their AUCs were mostly between those of the corresponding cranial and caudal original vertebral parameters, as shown in **Table 4** and **Supplementary Table 3**. This finding suggests that integration may help balance intervertebral differences and more stably reflect the overall imaging characteristics of the adjacent involved STB lesion unit.

Previous quantitative SPECT/CT studies on tumor related bone lesions have shown that SUVmax is an important parameter reflecting focal bone metabolic activity and may help differentiate bone metastases from benign or indeterminate bone lesions (12). Consistent with these findings, LBI_SUVmax_ showed the highest single parameter diagnostic performance among the integrated parameters in the present study, suggesting that integrated peak metabolic activity may provide useful information for distinguishing SM from STB. This finding may be explained by the different pathological bases of SM and STB. In SM, tumor cell infiltration and cytokine mediated bone remodeling can increase local osteoblastic activity and blood perfusion, thereby increasing ^99m^Tc-MDP uptake (32). In contrast, STB is characterized by granulomatous inflammation mediated by Th1 cells, macrophage activation, caseous necrosis, and coexistence of bone destruction and repair, which may lead to a more heterogeneous distribution of metabolic activity within the lesion (33, 34). Among the integrated CT attenuation parameters, LBI_CT HUmean_ also showed discriminatory value, although its performance was lower than that of metabolic parameters. This may be related to differences in structural bone changes between SM and STB. SM can present as osteoblastic, osteolytic, or mixed metastases, and different metastatic patterns may show variable CT attenuation and bone metabolic activity (35). In contrast, STB is often accompanied by infectious bone destruction, caseous necrosis, and inflammatory granulation tissue formation, which may reduce the average CT attenuation value within the lesion area (36). These structural differences may explain why LBI_CT HUmean_ has certain discriminatory value, although its single parameter diagnostic performance remains lower than that of metabolic parameters such as LBI_SUVmax_.

**Figure 5:**
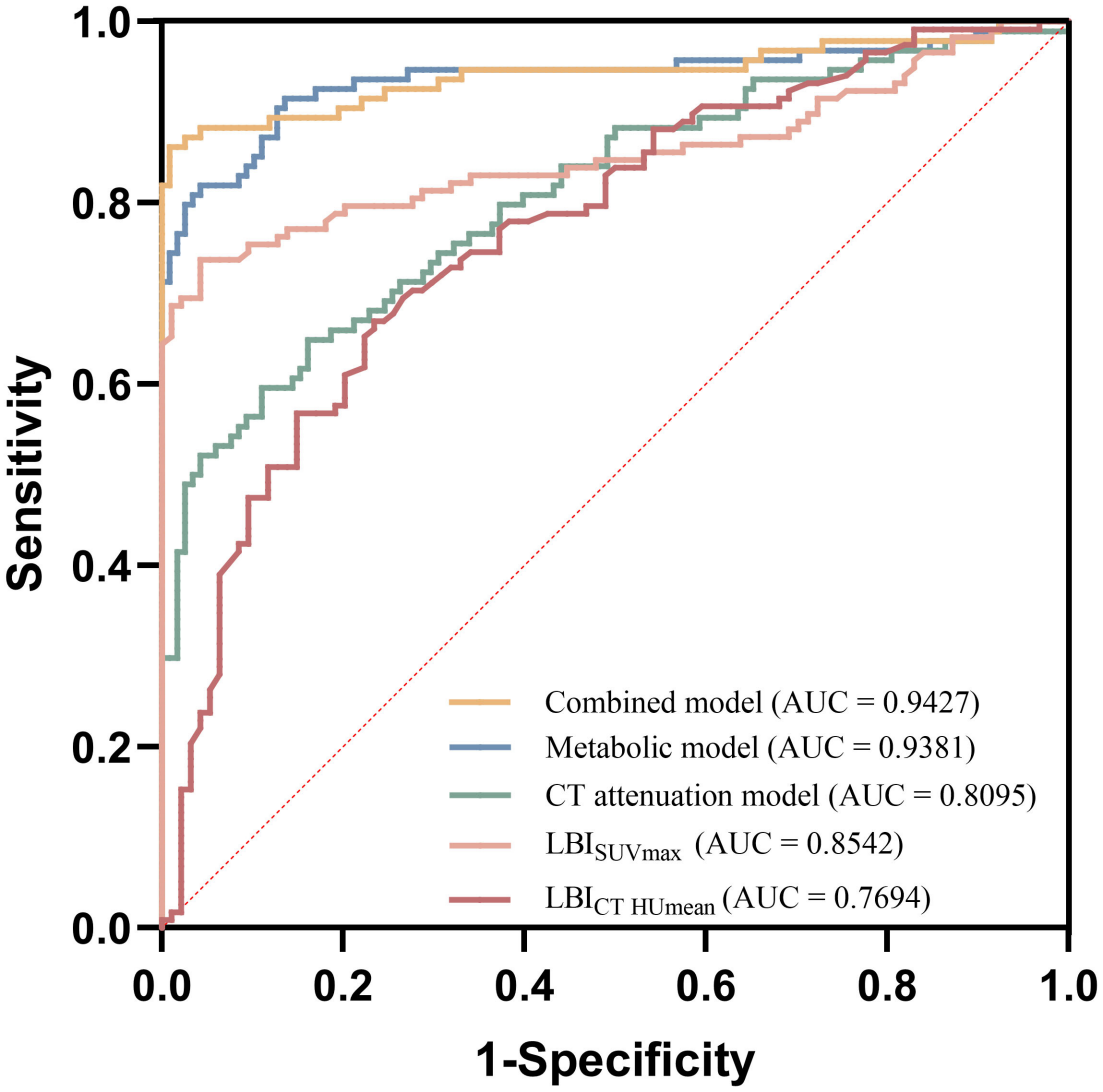
ROC curves for multivariable models and representative individual quantitative SPECT/CT parameters in differentiating STB from SM.

**Figure 6:**
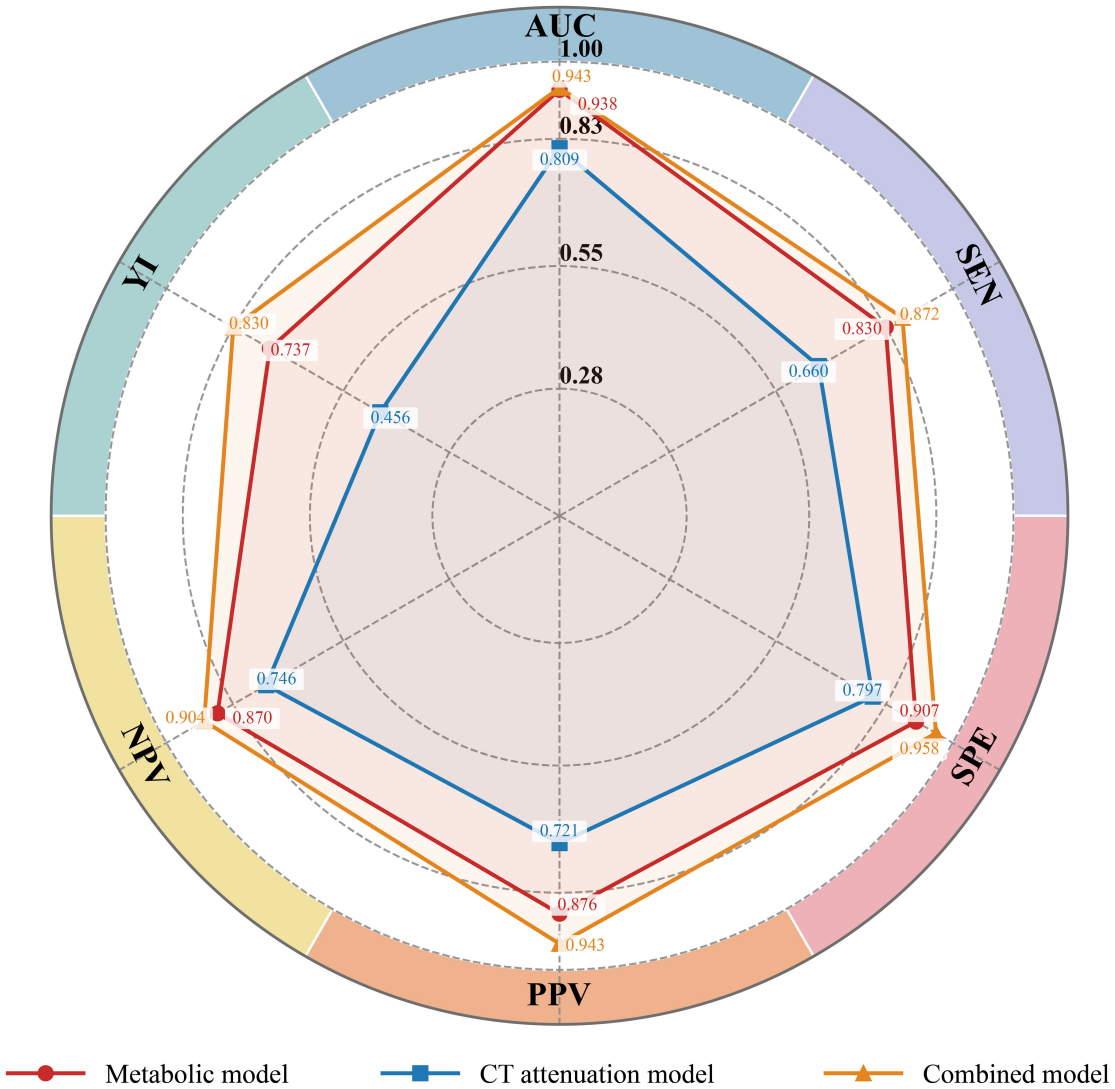
Radar chart of diagnostic performance metrics for the metabolic, CT attenuation, and combined models. Different colored lines represent the three models. Sensitivity, specificity, PPV, and NPV were plotted as proportions on a common 0–1 scale, whereas AUC and the Youden index were plotted as reported. AUC, area under the receiver operating characteristic curve; SEN, sensitivity; SPE, specificity; PPV, positive predictive value; NPV, negative predictive value; YI, Youden index.

Previous studies on the differentiation between STB and SM have mainly relied on clinical variables, laboratory markers, and MRI features. Cao et al. developed a scoring system using multivariable logistic regression, incorporating nocturnal pain, C-reactive protein (CRP), tumor markers, skip lesions, and intervertebral disc space destruction to distinguish between the two diseases (37). Their findings suggest that integrating clinical, laboratory, and conventional imaging features can improve the overall diagnostic assessment. Zhang et al. further developed a logistic regression model based on IVIM MRI quantitative parameters and reported that the combination of ADCfast and f achieved an AUC of 0.925, supporting the value of quantitative imaging parameter integration in differentiating STB from SM (38). In the present study, metabolic parameter, CT attenuation, and combined models were constructed based on quantitative ^99m^Tc-MDP SPECT/CT. The metabolic model achieved an AUC of 0.9381, which was higher than that of the CT attenuation model at 0.8095, as shown in **Figure 5**. This result was consistent with the superior performance of integrated metabolic parameters in the single parameter analysis. Although the combined model achieved the highest AUC, specificity, and Youden index, DeLong’s test showed no statistically significant difference in AUC between the combined model and the metabolic model, *p* = 0.422, as shown in **Supplementary Table 4**. This indicates that adding CT attenuation parameters did not significantly improve the overall discriminative ability beyond the metabolic model. Considering the higher specificity and Youden index of the combined model, CT attenuation parameters may provide complementary structural information, particularly in improving specificity and overall classification performance, as shown in **Figure 6**.

Decision curve analysis showed that the combined model and the metabolic model provided greater net benefit than the CT attenuation model within the prespecified threshold probability range, and the curves of the two models were relatively close, as shown in **Supplementary Figure 1**. This finding was consistent with the ROC and DeLong analyses. Although the combined model showed better performance in some diagnostic indicators, its overall gain over the metabolic model was limited, and its clinical net benefit still requires validation in an independent external cohort. Compared with conventional visual interpretation, this study incorporated SUV metabolic parameters, CT attenuation parameters, and the adjacent vertebral involvement pattern of STB into the same analytical framework. This approach allows STB and SM to be evaluated from both functional metabolic and structural attenuation perspectives. In particular, the construction of integrated parameters based on paired adjacent involved vertebrae in STB may better reflect the continuous adjacent involvement pattern of STB and provides a quantitative approach for the non invasive differentiation of STB and SM.

This study has several limitations. First, this was a retrospective single center study, and the included patients were from a single institution, which may have introduced selection bias in patient type and distribution. The applicability of the model to other institutions and patient populations needs further validation in multicenter prospective cohorts. Second, the SM group included heterogeneous primary tumor types. Osteoblastic, osteolytic, or mixed bone metastatic patterns from different tumors may influence ^99m^Tc-MDP uptake and CT attenuation parameters. Third, the analytical units were not completely identical between the two groups, as the STB group was analyzed using integrated parameters derived from two adjacent involved vertebrae, whereas the SM group was analyzed using one selected target metastatic vertebra. Although this strategy was based on the characteristic contiguous involvement pattern of STB, potential analytical-unit bias cannot be completely excluded. In addition, independent external validation was not performed; therefore, the proposed integrated parameters and diagnostic models should be further validated in larger multicenter prospective cohorts. Future studies should combine quantitative SPECT/CT parameters with MRI features, laboratory markers, and clinical variables to construct and validate more stable and clinically applicable diagnostic models in larger multicenter prospective cohorts.

In conclusion, quantitative ^99m^Tc-MDP SPECT/CT provides objective metabolic and CT attenuation information for the no invasive differentiation of STB and SM. Integrated parameters based on the adjacent vertebral involvement pattern of STB, particularly metabolic integrated parameters, may provide useful quantitative imaging information for differentiating these two diseases. These findings suggest that quantitative SPECT/CT may serve as an objective adjunct to conventional imaging when STB and SM show overlapping clinical and radiological features.

## Data Availability

The original contributions presented in the study are included in the article/**Supplementary Material**. Further inquiries can be directed to the corresponding authors.
