# Supplementary material for: Diagnostic value of quantitative SPECT/CT integrated parameters for differentiating spinal tuberculosis from spinal metastases
Source: Front Oncol. 2026 Jul 30;16:1890572. doi: 10.3389/fonc.2026.1890572 (PMC13467942; doi:10.3389/fonc.2026.1890572)
Supplement: Supplementary file 1 [file DataSheet1.docx]

Supplementary Material

Supplementary Table S1. Primary tumor types among patients with spinal metastases.

| **Primary tumor type** | **No. (%)** |
| --- | --- |
| Lung cancer | 52(44.07%) |
| Prostate cancer | 17(14.41%) |
| Breast cancer | 12(10.17%) |
| Gastric cancer | 6(5.08%) |
| Colon cancer | 5(4.23%) |
| Ureteral cancer | 8(6.78%) |
| kidney cancer | 4(3.39%) |
| Cervical cancer | 3(2.54%) |
| Thyroid cancer | 2(1.69%) |
| Nasopharyngeal carcinoma | 1(0.85%) |
| Unknown primary | 8(6.79%) |

Supplementary Table S2. Multivariable logistic regression coefficients and collinearity diagnostics for differentiating STB from SM target vertebrae.

| **Model** | **Variable** | **β coefficient** | **OR (95% CI)** | **VIF** |
| --- | --- | --- | --- | --- |
| Metabolic model | LBI_SUVmax_ | −0.898 | 0.408 (0.302–0.519) | 6.95 |
|  | SUVmin_int_ | 3.796 | 44.520 (12.270–237.900) | 2.50 |
|  | LBI_SUVmean_ | 0.067 | 1.069 (0.794–1.460) | 6.89 |
|  | TBU_AM_ | 0.001 | 1.001 (0.996–1.007) | 2.77 |
| CT attenuation model | LBI_CT HUmax_ | −0.003 | 0.997 (0.994–0.999) | 1.13 |
|  | CT HUmin_int_ | −0.046 | 0.955 (0.923–0.987) | 1.17 |
|  | LBI_CT HUmean_ | −0.013 | 0.987 (0.982–0.993) | 1.17 |
| Combined model | LBI_SUVmax_ | −0.783 | 0.457 (0.344–0.569) | 3.12 |
|  | SUVmin_int_ | 3.559 | 35.120 (9.867–187.600) | 2.52 |
|  | LBI_CT HUmax_ | −0.002 | 0.998 (0.995–1.001) | 1.18 |
|  | CT HUmin_int_ | −0.019 | 0.981 (0.933–1.031) | 1.26 |
|  | LBI_CT HUmean_ | −0.006 | 0.994 (0.987–1.001) | 1.29 |

Supplementary Table S3. ROC analysis of original parameters from upper and lower STB-involved vertebrae for differentiating STB from SM target vertebrae.

| **Category** | **Variable** | **AUC (95% CI)** | **Cutoff** | **Sensitivity (%)** | **Specificity (%)** | **Youden index** |
| --- | --- | --- | --- | --- | --- | --- |
| **Upper STB-involved vertebra vs. SM target vertebrae** | **Metabolic parameters** | | | | | |
|  | SUVmax | 0.8419  (0.7867–0.8971) | 21.47 | 94.68 | 71.19 | 0.659 |
|  | SUVmin | 0.5324  (0.4549–0.6099) | 2.76 | 100.00 | 16.10 | 0.161 |
|  | SUVmean | 0.749  (0.6838–0.8142) | 12.28 | 93.62 | 54.24 | 0.479 |
|  | TBU | 0.6624  (0.5895–0.7353) | 318.97 | 68.09 | 59.32 | 0.274 |
|  | **CT attenuation parameters** | | | | | |
|  | CT HUmax | 0.7106  (0.6414–0.7798) | 879.0 | 68.09 | 64.41 | 0.325 |
|  | CT HUmin | 0.5632  (0.4814–0.6449) | -33.0 | 36.17 | 87.29 | 0.235 |
|  | CT HUmean | 0.7522  (0.6854–0.8190) | 281.0 | 65.96 | 73.73 | 0.397 |
|  | | | | | | |
| **Lower STB-involved vertebra vs. SM target vertebrae** | **Metabolic parameters** | | | | | |
|  | SUVmax | 0.8679  (0.8185–0.9173) | 22.04 | 98.64 | 67.8 | 0.667 |
|  | SUVmin | 0.5533  (0.4759–0.6307) | 4.42 | 82.98 | 31.36 | 0.143 |
|  | SUVmean | 0.7687  (0.7068–0.8306) | 12.19 | 90.43 | 55.08 | 0.455 |
|  | **CT attenuation parameters** | | | | | |
|  | TBU | 0.8173  (0.7620–0.8725) | 247.0 | 68.09 | 78.81 | 0.469 |
|  | CT HUmax | 0.6832  (0.6117–0.7546) | 832.0 | 56.38 | 74.58 | 0.310 |
|  | CT HUmin | 0.5224  (0.4396–0.6053) | -30.0 | 38.30 | 78.87 | 0.171 |
|  | CT HUmean | 0.7796  (0.7182–0.8409) | 318.0 | 93.60 | 55.08 | 0.487 |

**Note:** Upper and lower vertebrae refer to the two adjacent involved vertebrae in each STB patient. SM values represent selected target metastatic vertebrae.

Supplementary Table S4. Pairwise comparisons of correlated ROC curves using the DeLong test.

| **Comparison** | **AUC 1** | **AUC 2** | **ΔAUC** | **SE** | **95% CI for ΔAUC** | ***Z* value** | ***P* value** |
| --- | --- | --- | --- | --- | --- | --- | --- |
| Combined model vs metabolic model | 0.9427 | 0.9381 | 0.0046 | 0.0057 | [−0.0066, 0.0158] | 0.802 | 0.422 |
| Combined model vs CT attenuation model | 0.9427 | 0.8095 | 0.1332 | 0.0273 | [0.0797, 0.1866] | 4.879 | 0.001 |
| Metabolic model vs CT attenuation model | 0.9381 | 0.8095 | 0.1286 | 0.0303 | [0.0691, 0.1880] | 4.239 | 0.001 |
| Combined model vs LBI_SUVmax_ | 0.9427 | 0.8542 | 0.0884 | 0.0290 | [0.0317, 0.1452] | 3.054 | 0.002 |
| Combined model vs LBI_CT HUmean_ | 0.9427 | 0.7694 | 0.1733 | 0.0325 | [0.1082, 0.2356] | 5.289 | 0.001 |

Supplementary Figure 1. Decision curve analysis of the combined, metabolic, and CT attenuation models for differentiating STB from SM. Dashed lines indicate treat-all and treat-none strategies.
